# Allergic Disease and Autoimmune Effectors Pathways

**DOI:** 10.1080/1044667031000137638

**Published:** 2002-09

**Authors:** Menachem Rottem, M. Eric Gershwin, Yehuda Shoenfeld

**Affiliations:** 1 Division of Allergy and Clinical Immunology Ha'Emek Medical Center Afula 18101 Israel; 2 Division of Rheumatology Allergy and Clinical Immunology TB 192 One Shields Avenue University of California at Davis Davis CA 95616 USA; 3 Department of Medicine “B” and Center for Autoimmune Diseases Sheba Medical Center Tel-Hashomer 52621 Israel

## Abstract

Allergy and autoimmunity result from dysregulation of the immune system. Until recently, it was generally accepted that the mechanisms that govern these disease processes are quite disparate; however, new discoveries suggest possible common pathogenetic effector pathways. This review illustrates the concomitant presentation of these conditions and the potential relationship or common mechanism in some cases, by looking at the key elements that regulate the immune response in both allergic and autoimmunite conditions: mast cells, antibodies, T cells, cytokines, and genetic determinants. The parallel appearance of allergic and autoimmune conditions in the some patients may reveal that such aberrations of the immune system have a common pathophysiologic mechanism. Mast cells, which play a key role in allergic reactions, and the wealth of inflammatory mediators they express, make it likely that they have profound effects on many autoimmune processes. Activation of protein kinases by inflammatory cytokines and environmental stresses may contribute to both allergic and autoimmune diseases. The presence of autoantibodies in some allergic conditions suggests an autoimmune basis for these conditions. Because of the central role T cells play in immune reactivity, the T-cell receptor (TCR) loci have long been considered important candidates for common disease susceptibility within the immune system such as asthma, atopy, and autoimmunity. Immunomodulation is the key to a successful treatment of allergic and autoimmune conditions.


*Developmental Immunology*, September 2002, Vol. 9 (3), pp. 161–167

### Allergic Disease and Autoimmune Effectors Pathways

MENACHEM ROTTEM[a,*], M. ERIC GERSHWIN[b,†] and YEHUDA SHOENFELD[c,‡]

[a]*Division of Allergy and Clinical Immunology, Ha'Emek Medical Center, Afula, 18101, Israel;* [b]*Division of Rheumatology, Allergy and Clinical Immunology, TB 192 One Shields Avenue, University of California at Davis, Davis, CA 95616, USA;* [c]*Department of Medicine "B" and Center for Autoimmune Diseases, Sheba Medical Center, Tel-Hashomer 52621, Israel*

Allergy and autoimmunity result from dysregulation of the immune system. Until recently, it was generally accepted that the mechanisms that govern these disease processes are quite disparate; however, new discoveries suggest possible common pathogenetic effector pathways. This review illustrates the concomitant presentation of these conditions and the potential relationship or common mechanism in some cases, by looking at the key elements that regulate the immune response in both allergic and autoimmunite conditions: mast cells, antibodies, T cells, cytokines, and genetic determinants. The parallel appearance of allergic and autoimmune conditions in the some patients may reveal that such aberrations of the immune system have a common pathophysiologic mechanism. Mast cells, which play a key role in allergic reactions, and the wealth of inflammatory mediators they express, make it likely that they have profound effects on many autoimmune processes. Activation of protein kinases by inflammatory cytokines and environmental stresses may contribute to both allergic and autoimmune diseases. The presence of autoantibodies in some allergic conditions suggests an autoimmune basis for these conditions. Because of the central role T cells play in immune reactivity, the T-cell receptor (TCR) loci have long been considered important candidates for common disease susceptibility within the immune system such as asthma, atopy, and autoimmunity. Immunomodulation is the key to a successful treatment of allergic and autoimmune conditions.

*Keywords*: Allergy; Autoimmunity; Asthma; IgE; Urticaria

### INTRODUCTION

Malfunction of the immune system is known in three main conditions: immunodeficiency, allergy, and autoimmunity. These states usually appear separately. Two of the conditions, immunodeficiency and autoimmunity, sometime appear together, such as autoimmunity in IgA deficiency or in common variable immunodeficiency. The appearance of autoimmunity and allergies has not been well appreciated. Recently, there are reports on the appearance of allergies concomitantly with autoimmunity, but the relationship is poorly understood. Allergy and autoimmunity are two potential outcomes of dysregulated immunity. Both are characterized by localized inflammation that leads to the injury and/or destruction of target tissues. Until recently, it was generally accepted that the mechanisms that govern these disease processes are quite disparate; however, new discoveries suggest possible pathogenetic linkage. In this review we shall summarize and discuss the concomitant presentation of these conditions and the potential relationship or common mechanism, and we will do so by looking at the key elements that regulate the immune response in allergy and autoimmunity.

### MAST CELLS IN ALLERGIC AND AUTOIMMUNE DISEASES

Mast cells are distributed widely throughout mammalian tissues, especially the perivascular spaces and connective tissues in the skin, respiratory and gastrointestinal tracts. Cross-linkage of the high affinity IgE receptors elicits the release of preformed granules including histamine, neutral proteases, cytokines and proteoglycans (Stevens and Austen, 1989). There is also a rapid synthesis and release of prostaglanduns and leukotriens. Activated mast cells also synthesize and secrete several proinflammatory, chemoattractive, and immunomodulatory cytokines over a period of several hours. While the role of mast cells in allergic diseases is well recognized, it was not until recently that knowledge has been gathered on their physiologic role and their role in non-allergic diseases and non-IgE mediated stimuli (Stevens and Austen, 1989).

*Corresponding author. Tel.: +972-(0)58-617823. Fax: +972-(0)4-6593629. E-mail: menachem@rottem.net
†The Jack and Donald Chair Professor of Medicine.
‡Incumbent of the Laura Schwarz-Kipp Chair for Research of Autoimmune Diseases, Tel-Aviv University, Tel-Aviv, Israel.

ISSN 1044-6672 print © 2002 Taylor & Francis Ltd
DOI: 10.1080/1044667031000137638

Mast cells play a pivotal role in allergy and inflammation. There is a prominence of lesional mast cells in multiple sclerosis, (MS) (Ibrahim *et al.*, 1996), cardiomyopathy (Arbustini *et al.*, 1997), congestive heart failure (Hara *et al.*, 2002), rheumatoid arthritis, (RA) (Gotis-Graham and McNeil, 1997; Woolley and Tetlow, 2000; Lee *et al.*, 2002), liver disease (Matsunaga *et al.*, 1999), and fibrotic lung conditions (Pesci *et al.*, 1993). Previous studies have revealed that autoantibodies, complement components, and Fc receptors each participate in the pathogenesis of erosive arthritis in $K/B \times N$ mice. However, it is not known which cellular populations are responsive to these inflammatory signals. Two strains of mice deficient in mast cells, $W/W^v$ and $Sl/Sl^d$, are resistant to development of joint inflammation and susceptibility is restored in the $W/W^v$ strain by mast cell engraftment. Thus, mast cells may function as a cellular link between autoantibodies, soluble mediators, and other effector populations in inflammatory arthritis (Lee *et al.*, 2002). Mast cell-deficient mice exhibit significantly reduced disease severity compared to wild-type littermates in a murine model of MS and drugs that block mast cell function can improve clinical symptoms in this model. In addition, gene microarray analysis has revealed that the expression of several mast cell-specific genes is increased in the central nervous system plaques of MS patients. Although well established as effector cells in allergic inflammation, the location of mast cells and the wealth of inflammatory mediators they express make it likely that they have profound effects on many other autoimmune processes (Robbie-Ryan and Brown, 2002).

### AUTOANTIBODIES IN ALLERGIC AND AUTOIMMUNE DISEASES

The allergic response to common environmental allergens has been regarded as an important mechanism in the development of airway inflammation of patients with asthma. The idea of a possible involvement of autoimmunity in the pathogenesis of non-allergic asthma has been proposed by few studies (Lidor *et al.*, 1980).

Atopic individuals with asthma, allergic rhinitis or atopic eczema have impaired sensitivity to beta-adrenergic agents. Antibodies directed toward the beta adrenergic receptor were found in sera of some patients with asthma (Harrison *et al.*, 1982) suggesting that autoantibodies to beta-receptors play a pathogenetic role in asthma and related disorders. The serum gamma-globulin fraction from patients with allergic bronchial asthma was found to inhibit the positive chronotropic action of the beta 2-selective adrenergic agonist, but not of beta 1-adrenergic agonist (Wallukat and Wollenberger, 1991). The inhibitory immunoglobulins of the patients with asthma were mainly autoantibodies of the IgG isotype, capable of cross-reacting with chronotropic beta 2-adrenergic receptors on the cultured rat cardiomyocytes. These findings provide support for the notion that autoimmunity plays a role in the impairment of beta-adrenergic responsiveness in patients with allergic bronchial asthma. Similar studies were performed to reveal the role of antibodies on bronchial epithelium. Serum samples from healthy controls, patients with atopic asthma, patients with non-atopic asthma, and patients with systemic lupus erythematosus were studied for the presence of circulating autoantibodies to cultured human bronchial epithelial cell using enzyme-linked immunosorbent assay (Nahm *et al.*, 2001). Levels of IgG autoantibodies to bronchial epithelial cell were significantly higher in patients with non-atopic asthma and systemic lupus erythematosus than in healthy controls and patients with atopic asthma. These circulating IgG autoantibodies were further studied by immunoblot analysis (Nahm *et al.*, 2002). IgG autoantibodies to the 49-kD bronchial epithelial cell antigen were detected in 43% of patients with non-allergic, 11% of patients with allergic asthma, 10% of patients with systemic lupus erythematosus, and 9% healthy volunteers ($P < 0.005$). The 49-kD auto-antigen was purified and identified as cytokeratin 18 by amino acid sequencing. In this study cytokeratin 18 was identified as a bronchial epithelial autoantigen associated with non-allergic asthma. Taken together, the presence of autoantibodies to beta adrenergic receptors and bronchial epithelium in patients with atopic and non-atopic asthma, respectively, demonstrate autoimmune phenomena in these conditions.

Patients suffering from atopic dermatitis (AD) frequently display IgE autoantibody reactivity to human proteins expressed in cell lines of different histogenetic origins. Molecular cloning and *in situ* staining experiments revealed that certain IgE-reactive autoantigens were expressed preferentially in target organs of atopy while others could be detected in various tissues and cell types (Seiberler *et al.*, 1999). Serum IgE from AD patients was used to investigate the distribution of autoantigens in subcellular fractions of the human epithelial cell line A431 and in human tissue specimens. Results showed that IgE-reactive autoantigens can be detected in the nuclear > microsomal > mitochondrial > cytoplasmic fraction of A431 cells as well as in a variety of human tissue specimens (brain, bone, intestine, liver, lung, muscle, skin, uterus) and effector cells of atopy (basophils, mast cells, T cells). If IgE autoreactivity plays a pathogenetic role in severe forms of atopy, organ-specific manifestations (e.g. AD) may result from the transport to and deposition of IgE-reactive autoantigens in certain target organs (skin) rather than from a preferential expression of the autoantigens in the affected tissues.

Schnitzler's syndrome is a rare disease characterized by chronic urticaria, monoclonal IgM, and clinical and laboratory signs of inflammation. In a subset of patients, the urticarial lesions cause pruritus. However, the pathophysiology of the disease and the biochemical basis of urticaria are not known. Sperr *et al.* (2000) described a subclass-specific IgG reactivity against microvascular endothelial cells and mast cells indicating Th1 autoimmunity in a patient with Schnitzler's syndrome.

Whether such autoantibodies are recurrently produced in patients with Schnitzler's syndrome and play a role in the pathophysiology of the disease remains to be determined.

Szczeklik *et al.* (1995) assessed the autoimmune status of 185 adult patients with bronchial asthma and 46 healthy subjects of similar sex and age. The patients were divided into groups with: aspirin-induced asthma (AIA), intrinsic asthma with good aspirin tolerance, and atopic asthma. Antinuclear antibodies (ANA) at a titer of > or = 1:40 were present in the serum of 55% of the patients with AIA, 41% of those with intrinsic asthma, 39% of those with atopic asthma, and 11% of the healthy subjects, with the difference between each patient group and the healthy subjects being statistically significant ($P < 0.05$). The fluorescence staining pattern of ANA on Hep-2 cells was mainly speckled, but the precise antigen specificity of the antibodies could not be identified with reference sera against extractable nuclear antigens. None of the subjects exhibited anti-double stranded deoxyribonucleic acid (anti-ds-DNA) or anti-neutrophil cytoplasmic antibodies (ANCA). Positive ANA were associated with signs of complement activation, the presence of rheumatoid factor, and circulating immune complexes. Clinical signs of autoimmunity, evidenced by rheumatic symptoms, cold sensitivity, and Raynaud's phenomenon, were more common among the patients who tested positively for ANA. These results should, however, be interpreted cautiously, since ANA positivity in 1:40 dilution can be found in a significant number of healthy individuals (Tan *et al.*, 1997). Based on 15 international laboratories experienced in performing ANA tests, Tan *et al.* revealed that the normal population was ANA positive in 31.7% of individuals at 1:40 serum dilution, 13.3% at 1:80, 5.0% at 1:160, and 3.3% at 1:320. They concluded that it is recommended that laboratories performing immunofluorescent ANA tests should report results at both the 1:40 and 1:160 dilutions, and should supply information on the percentage of normal individuals who are positive at these dilutions. A low-titer ANA is not necessarily insignificant. ANA assays can be a useful discriminant in recognizing certain disease conditions, but can create misunderstanding when the limitations are not fully appreciated. The assessment of autoimmunity may help in better characterizing patients with asthma and understanding various symptoms of the disease. Any causal relationship between asthma and autoimmunity remains to be established.

### T-CELL DYSREGULATION IN ALLERGIC AND AUTOIMMUNE DISEASES

Regulatory T cells (also referred to as suppressor T cells) are important components of the homeostasis of the immune system, as impaired regulatory T cell activity can cause autoimmune diseases and atopy. CD8+ T cells are known to mediate delayed-type hypersensitivity (DTH) responses in allergic contact dermatitis, drug eruptions, asthma, and autoimmune diseases. DTH is defined as the recruitment of T cells into tissues to be activated by antigen-presenting cells to produce cytokines that mediate local inflammation. This inflammatory effector capability of CD8+ cytotoxic T cells was previously poorly recognized, but there is now considerable evidence that inflammatory diseases may be mediated by CD8+ DTH (Kalish and Askenase, 1999).

The cause of asthma, which has been linked to a chronic, T-cell-mediated bronchial inflammation, remains unclear. A number of other T-lymphocyte-mediated, chronic inflammatory disorders have been associated with autoimmunity and there are data indicating that autoimmune phenomena might also be present in asthma. Arnold *et al.* (2000) tested the hypothesis that allergic and intrinsic asthma might be associated with an increase in lymphocytes producing perforin, a cytotoxic molecule produced by lymphocytes that has been implicated in the pathogenesis of autoimmune diseases, by comparing the expression of intracellular perforin in peripheral blood lymphocytes of patients with extrinsic asthma compared to patients with intrinsic asthma and healthy control subjects. The percentage of perforin-positive total lymphocytes was significantly elevated in patients with allergic as well as intrinsic asthma when compared with normal control subjects. Analysis of lymphocyte subpopulations also revealed a significant increase in the percentage of CD3(+), CD4(+), CD8(+), and CD56(+) cells expressing perforin in allergic asthma and a significant increase in the percentage of CD4(+) and CD56(+) cells in intrinsic asthma when compared with healthy control subjects. Perforin expression in CD4(+) cells in intrinsic asthma was also significantly elevated compared with allergic asthma. Thus, allergic and intrinsic asthma is associated with increased expression of perforin in T-lymphocyte subsets.

The responses to allergens are typically of the TH2 phenotype, with IL-4, IL-5, and IL-13 playing roles in IgE isotype switching and eosinophil recruitment to the site of inflammation. CD4+ CD25+ T cells have been shown to regulate TH2 immune responses; however, their role in control of allergic responses has yet to be defined. TH1 cells exclusively produce IL-2 and IFN-γ, and expression of IL-1, IL-3, IL-10 and granulocyte-macrophage colony-stimulating factor (GM-CSF) are shared between TH1 and TH2 subsets. TH1 subset, by virtue of IFN-γ secretion, confers a protective cell-mediated response to both intracellular and extracellular bacterial pathogens. In this case, IFN-γ is required for the oxidative burst and delayed-type. This type of response would be ineffectual to pathogens such as helminthes because these large organisms are resistant to phagocytosis and intracellular oxidative bursts. Thus, TH2 cells in response to helminth parasites mediate a protective type II response. In this situation, TH2 secretion of IL-4 elicits B-cell production of helminth antigen-specific IgE and secreted IgA, and IL-5 induces the recruitment and activation of eosinophils to sites of. Clearly, host-pathogen interactions have provided a necessary selective pressure for the evolution of TH subsets. Rheumatic diseases have divergent

TH1/TH2 polarity. Ankylosing spondylitis (AS) for example exhibit TH2 tendency, while RA exhibit Th1 cytokine profile. Atopic disorders are decreased in RA but only slightly and non-significantly increased in AS (Rudwaleit *et al.*, 2002). This may imply that atopy confers some protection from RA but only little if any susceptibility to AS. It may further indicate that the cytokine deviation towards an impaired TH1 pattern in AS is less strong than the cytokine deviation towards TH1 in RA, a finding which may affect future therapeutic approaches. There is a significantly lower incidence of allergic diseases in SLE patients, especially those who have an allergic family history, when compared with the controls (Sekigawa *et al.*, 2002). These findings may be related to the immunological similarities and differences between SLE and various allergic diseases. Suppressor T cells play important roles in the regulation of immune responses and the mediation of dominant immunologic tolerance. Studies of suppressor T-cell function were recently identified as a minor fraction (approximately 10%) of CD4(+) T cells that coexpress CD25. CD4(+) CD25(+) T cells have been shown to play a critical role in the prevention of organ- specific autoimmunity and allograft rejection (McHugh and Shevach, 2002). Because tumor antigens are self-antigens, it is not surprising that CD4(+) CD25(+) T cells also inhibit the induction of tumor immunity. Although CD4(+) CD25(+) T cells are capable of inhibiting TH2 responses, their role in the suppression of allergic responses has not been firmly established. Depending on the desired immune response, enhancement or restraint of suppressor T-cell function might be required.

Because of the central role T cells play in immune reactivity, the T-cell receptor (TCR) loci have long been considered important candidates for common disease susceptibility within the immune system such as asthma, atopy, and autoimmunity.

Over the past two decades, hundreds of sequence variations known as single nucleotide polymorphism (SNPs) in the TCR loci have been identified. Most studies have focused on defining SNPs in the variable gene segments which are involved in antigenic recognition. On average, the coding sequence of each TCR variable gene segment contains two SNPs, with many more found in the 5′, 3′ and intronic sequences of these segments (Mackelprang *et al.*, 2002). Meaningful association studies in the TCR loci will require the analysis and typing of large marker sets to fully evaluate the role of TCR loci in common disease susceptibility in human populations. Inherited abnormalities in either coding or regulatory regions of TCR genes may predispose to aberrant T-cell function and autoimmune disease (Kay, 1996).

### CYTOKINE DYSREGULATION IN ALLERGIC AND AUTOIMMUNE DISEASES

Multicellular organisms have three well-characterized subfamilies of mitogen-activated protein kinases (MAPKs) that control a vast array of physiological processes (Johnson and Lapadat, 2002). These enzymes are regulated by a characteristic phosphorelay system in which a series of three protein kinases phosphorylate and activate one another. The extracellular signal-regulated kinases (ERKs) function in the control of cell division, and inhibitors of these enzymes are being explored as anticancer agents. The c-Jun amino-terminal kinases (JNKs) are critical regulators of transcription, and JNK inhibitors may be effective in control of RA. The p38 MAPKs are activated by inflammatory cytokines and environmental stresses and may contribute to diseases like asthma and autoimmunity.

### GENETIC DETERMINANTS IN ALLERGY ASTHMA AND AUTOIMMUNE DISEASES

The genetic basis of atopic diseases is represented by a complex network of interacting genes or common genetic variants rather than a few disease-causing mutations. Polymorphism within IL-4R alpha, which is associated with asthma and atopy, is also associated with kidney systemic lupus erythematosus and Crohn's disease (Heinzmann and Deichmann, 2001). On the other hand, there is a lower incidence or at least no increased risk of allergic diseases in SLE patients, especially those who had an allergic family histories, when compared with the controls (Morton *et al.*, 1998; Sekigawa *et al.*, 2002).

### CHRONIC URTICARIA AND AUTOIMMUNITY

Chronic urticaria deserves special consideration because few elements may be invovled in its pathogenesis. Chronic idiopathic urticaria (CIU) is a common and frustrating disorder. A subset of patients with CIU has been classified as autoimmune on the basis of two main findings: association with thyroid autoimmunity and anti-IgE and/or anti-IgE receptor antibodies (Leznoff *et al.*, 1983). CIU occurs in persons who do not have an increased incidence of AD, allergic rhinitis, or asthma compared with the incidence of these disorders in persons without CIU. Their IgE level, as a group, is within normal limits. CIU does not appear to be an allergic reaction in the classic sense, because IgE antibody is not involved and no external allergen is needed to initiate or perpetuate the process. There is growing evidence that some cases of CIU are associated with thyroid autoimmunity. Patients with CIU have an increased frequency of Hashimoto thyroiditis with the presence of antibodies to thyroglobulin or a microsomal-derived antigen (peroxidase), even in euthyroid patients. No broad non-specific autoimmunity is present. Antibodies reactive with $Fc_{\epsilon}RI$, the high-affinity IgE receptor are found in sera of 10–40% of patients with CIU. A smaller subset of patients with CIU lack anti-$Fc_{\epsilon}RI$ antibodies and have anti-IgE antibodies that cross-link $Fc_{\epsilon}RIs$ occupied by IgE. There are minorities of CIU

patients that lack such antibodies, but can degranulate basophils by a serum factor that has not yet been determined. Most patients with anti IgE antibodies have an IgG antibody directed against the 34 kD α subunit of the IgE receptor. There are no data to suggest that any of the anti thyroid antibodies is pathogenic in terms of CIU, and most likely these are associated, parallel, autoimmune events.

While the pathogenesis of CIU is not completely understood, mast cell activation is thought to be of major importance. Antibodies reactive with FcεRI, the high-affinity IgE receptor, were found in sera of 10–40% of patients with CIU (Gruber *et al.*, 1988; Leznoff and Sussman, 1989). These antibodies have the potential to cross-link $Fc_{\epsilon}$RIs on mast cells and basophils. This leads to the release of mediators, chemokines, and cytokines. A smaller subset of patients with CIU lack anti-FcεRI antibodies and have anti-IgE antibodies that cross-link $Fc_{\epsilon}$RIs occupied by IgE. Such antibodies were not specific for CIU, however, and were detected in patients with AD. A large number of patients with CIU have a positive autologous skin test (Grattan *et al.*, 1991). Most members of subpopulation of positive patients studied in much greater detail were found to have an IgG antibody directed to the α subunit of the IgE receptor (Hide *et al.*, 1993). Patients with CIU and Hashimoto's thyroiditis but not patients without Hashimoto's thyroiditis had anti-FcεRI antibodies in their sera that could induce degranulation of normal basophils (Kandeel *et al.*, 2001). Some sera from patients with chronic urticaria and Hashimoto's thyroiditis caused degranulation of normal basophils in the absence of anti-$Fc_{\epsilon}$RI antibodies. The factor causing basophil degranulation was not determined (Kandeel *et al.*, 2001). These results showed that Hashimoto's thyroiditis likely represents a marker of autoimmunity, rather than being a direct cause for CIU. Immunoblot analysis demonstrated that anti-IgE receptor antibodies bind to the 34-kD cloned α subunit (Fiebiger *et al.*, 1995). Recently a case of CIU was described in association with Graves' disease, where anti $Fc_{\epsilon}$RI antibodies were detected in addition to TSHR antibodies (Irani *et al.*, 2001). This report showed the coexistence of these two conditions was mediated by a different anti receptor antibody. Komiya *et al.* (2001) analyzed the relationship between serum IgE concentrations and the remission or recurrence of Graves' disease. Remission was found in 48.8% of patients with elevated IgE concentrations after 18 months of treatment with methimazole (MMI), as opposed to 80.3% of patients with normal concentrations ($P = 0.0014$). The rate of long-standing remission was lower in patients with elevated than normal IgE concentration (34.1 vs. 69.7%, $P = 0.0007$). Serum levels of interleukin (IL)-13 were also analyzed. Although IL-13 was not detected in all patients, the detection rate was higher in patients without remission and in those with recurrence than in those with long-standing remission ($P = 0.0012$). Thus, the elevation of IgE and the higher detection rate of IL-13 are associated with both remission and recurrence of Graves' disease.

Previous studies have found that 30–40% of hyperthyroid patients with Graves' disease have an elevation of serum IgE concentrations ($\geq 170$ IU/ml) (Sato *et al.*, 1999). In contrast, the prevalence of IgE elevation was significantly less in autoimmune Hashimoto's thyroiditis (Komiya *et al.*, 2001). Interestingly, decreases in TSH-binding inhibiting immunoglobulin (TBII) and thyroid-stimulating antibody (TSAb) in response to antithyroid drugs were less pronounced in patients with than without IgE elevation (Sato *et al.*, 1999). The role on IgE in autoimmune Graves' disease is not known. The most plausible explanation is that IL-13 is secreted from Th-2 cells and has functions to stimulate B cells to secrete TBII, TSAb, and IgE. Additional studies are required to confirm this concept. An elevation in IgE concentration is thought to be linked with hereditary abnormalities (Hopkin, 1990; Marsh, 1997). Human IL-4 operates through the IL-4 receptor (IL4R), thereby modulating IgE production and Th-2 inflammatory reaction (Hakonarson *et al.*, 1999). As is the case with IL-4, IL-13 operates through the IL-13R to stimulate IgE production and Th-2 inflammatory reactions. Moreover, IL-13R and IL4R share a common component in IL4Rα (Zurawski and de Vries, 1994) that is crucial for IL-4 (or IL-13) binding and signal transduction de Vries, 1998. Gain-in-function mutations in IL4Rα have been reported to be associated with atopy or asthma patients with elevated IgE concentrations (Khurana-Harshey *et al.*, 1997; Mitsuyasu *et al.*, 1998). These might be present in Graves' patients with elevated IgE, even if peripheral IL-13 or IL-4 were within normal range or not detected.

### IMPLICATIONS FOR THERAPY—IMMUNOMODULATION

Treatment of mast cells disease usually consists of anti histamines and local application of glucocorticoids. Future therapy potentially may include anti SCF antibodies, directed against the main cytokine which controls mast cell viability and survival (Table I).

Induction of long-term, antigen-specific immunologic unresponsiveness holds great promise for the treatment of many immune system-mediated diseases, including asthma, allergies, autoimmune diseases, and transplant rejection. Unlike current immunosuppressive treatments, immunologic tolerance therapies would affect only the undesired immune responses, leaving protective immunity intact (Hackett and Dickler, 1999). A variety of approaches to immunologic tolerance induction are being taken, reflecting the molecular and cellular complexity of immune system activation and regulation. Possible approaches include the following: interference with costimulatory signals in T-cell induction, TCR antagonism by altered peptides, exploitation of antigen-induced apoptosis to eliminate undesired T cells, opposition of inflammation by the induction of regulatory cytokines, induction of transplant tolerance by mixed chimerism, and deviation from deleterious allergic

TABLE I Pathogenic factors, conditions and potential treatments

| Pathogenetic factor | Disease known to be affected | | Potential treatment | |
|---|---|---|---|---|
| | Allergic | Autoimmune | Currently available* | Future |
| Mast cell | Pivotal role in all allergic conditions, including early (immediate) phase and late (inflammatory) phase | Multiple sclerosis, cardiomyopathy, congestive heart failure and myocardial infarction, rheumatoid arthritis, liver disease, fibrotic lung disease | Antihistamines, mast cell stabilizers | Anti stem cell factor (SCF) antibodies |
| Autoantibodies | Asthma, chronic urticaria | Pivotal role in numerous autoimmune conditions such as Hashimoto's thyroiditis, Graves' disease, myasthenia gravis, hemolytic anemia, ITP | Plasmapheresis IVIG (anti idiotype) | Anti IgE antibodies |
| T cell and cytokine dysregulation | Most allergic conditions, allergic contact dermatitis, asthma, drug eruption, TH2 type conditions | Pivotal role in numerous autoimmune conditions such as SLE, TH1 and TH2 type conditions | Immunotherapy, anti TNF alpha antibodies | Immunomodulation by DNA vaccination, anti cytokine antibodies (IL-4, IL-5, etc.) |

* Potential or existing treatment directed toward the pathogenetic cause and not including specific treatment for each condition or organ failure.

antibody responses by use of immunostimulatory DNA sequences. These multifaceted approaches are strongly supported by knowledge of basic immune mechanisms, which should facilitate the rational development of these therapies for controlling immune-mediated diseases. Therefore, immunologic or pharmacologic manipulation of regulatory T-cell populations represents an important future approach to immunotherapy of a wide range of immune responses.

### *References*

Arbustini, E., Gavazzi, A., Dal Bello, B., Morbini, P., Campana, C., Diegoli, M., Grasso, M., Fasani, R., Banchieri, N., Porcu, E., Pilotto, A., Ponzetta, M., Bellini, O., Lucreziotti, S. and Vigano, M. (1997) "Ten-year experience with endomyocardial biopsy in myocarditis presenting with congestive heart failure: frequency, pathologic characteristics, treatment and follow-up", *G. Ital. Cardiol.* **27**, 209–223.

Arnold, V., Balkow, S., Staats, R., Matthys, H., Luttmann, W. and Virchow, J.C., Jr. (2000) "Increase in perforin-positive peripheral blood lymphocytes in extrinsic and intrinsic asthma", *Am. J. Respir. Crit. Care Med.* **161**, 182–186.

Fiebiger, E., Maurer, D., Holub, H., Reininger, B., Hartmann, G., Woisetschlager, M., Kinet, J.P. and Stingl, G. (1995) "Serum IgG autoantibodies directed against the α chain of $Fc_{\epsilon}RI$: a selective marker and pathogenic factor for a distinct subset of chronic urticaria patients", *J. Clin. Investig.* **96**, 2606–2612.

Gotis-Graham, I. and McNeil, H.P. (1997) "Mast cell responses in rheumatoid synovium. Association of the MCTC subset with matrix turnover and clinical progression", *Arthritis Rheum.* **40**, 479–489.

Grattan, C.E.H., Francis, D.M., Hide, M. and Greaves, M.W. (1991) "Detection of circulating histamine-releasing autoantibodies with functional properties of anti IgE in chronic urticaria", *Clin. Exp. Allergy* **21**, 695–704.

Gruber, B.L., Baeza, M., Marchese, M., Agnello, V. and Kaplan, A.P. (1988) "Prevalence and functional role of anti-IgE autoantibodies in urticarial syndromes", *J. Investig. Dermatol.* **90**, 213–217.

Hackett, C.J. and Dickler, H.B. (1999) "Immunologic tolerance for immune system-mediated diseases", *J. Allergy Clin. Immunol.* **103**, 362–370.

Hakonarson, H., Maskeri, N., Carter, C. and Grunstein, M.M. (1999) "Regulation of TH1- and TH2-type cytokine expression and action in atopic asthmatic sensitized airway smooth muscle", *J. Clin. Investig.* **103**, 1077–1087.

Hara, M., Ono, K., Hwang, M.W., Iwasaki, A., Okada, M., Nakatani, K., Sasayama, S. and Matsumori, A. (2002) "Evidence for a role of mast cells in the evolution to congestive heart failure", *J. Exp. Med.* **195**, 375–381.

Harrison, L.C., Callaghan, J., Venter, J.C., Fraser, C.M. and Kaliner, M.L. (1982) "Atopy, autonomic function and beta-adrenergic receptor autoantibodies", *Ciba Found. Symp.* **90**, 248–262.

Heinzmann, A. and Deichmann, K.A. (2001) "Genes for atopy and asthma", *Curr. Opin. Allergy Clin. Immunol.* **1**, 387–392.

Hide, M., Francis, D.M., Grattan, C.E.H., Hakimi, J., Kochan, J.P. and Greaves, M.W. (1993) "Autoantibodies against the high affinity IgE receptor as a cause of histamine release in chronic urticaria", *N. Engl. J. Med.* **328**, 1599–1604.

Hopkin, J. (1990) "A genetic approach to atopy", *Eur. Respir. J.* **3**, 851–852.

Ibrahim, M.Z., Reder, A.T., Lawand, R., Takash, W. and Sallouh-Khatib, S. (1996) "The mast cells of the multiple sclerosis brain", *J. Neuroimmunol.* **70**, 131–138.

Irani, C., Gordon, N.D., Zweiman, B. and Levinson, A.I. (2001) "Chronic urticaria/angioedema and Graves' disease: coexistence of 2 antireceptor antibody-mediated diseases", *J. Allergy Clin. Immunol.* **108**, 874.

Johnson, G.L. and Lapadat, R. (2002) "Mitogen-activated protein kinase pathways mediated by ERK, JNK, and p38 protein kinases", *Science* **298**, 1911–1912.

Kalish, R.S. and Askenase, P.W. (1999) "Molecular mechanisms of CD8+ T cell-mediated delayed hypersensitivity: implications for allergies, asthma, and autoimmunity", *J. Allergy Clin. Immunol.* **103**, 192–199.

Kandeel, A.A., Zeid, M., Helm, T., Lillie, M.A., Donahue, E. and Ambrus, J.L., Jr. (2001) "Evaluation of chronic urticaria in patients with Hashimoto's thyroiditis", *J. Clin. Immunol.* **21**, 335–347.

Kay, R.A. (1996) "TCR gene polymorphisms and autoimmune disease", *Eur. J. Immunogenet.* **23**, 161–177.

Khurana-Harshey, G.K., Friedrich, M.F., Esswein, L.A., Thomas, M.L. and Chatila, T.A. (1997) "The association of atopy with a gain-of-function mutation in the α subunit of the interleukin-4 receptor", *N. Engl. J. Med.* **337**, 1720–1725.

Komiya, I., Yamada, T., Sato, A., Kouki, T., Nishimori, T. and Takasu, N. (2001) "Remission and recurrence of hyperthyroid Graves' disease during and after methimazole treatment when assessed by IgE and interleukin 13", *J. Clin. Endocrinol. Metab.* **86**, 3540–3544.

Lee, D.M., Friend, D.S., Gurish, M.F., Benoist, C., Mathis, D. and Brenner, M.B. (2002) "Mast cells: a cellular link between autoantibodies and inflammatory arthritis", *Science* **97**, 1689–1692.

Leznoff, A. and Sussman, G.L. (1989) "Syndrome of idiopathic chronic urticaria and angioedema with thyroid autoimmunity: a study of 90 patients", *J. Allergy Clin. Immunol.* **84**, 66–71.

Leznoff, A., Josse, R.G., Denberg, J. and Dolovich, J. (1983) "Association of chronic urticaria and angioedema with thyroid autoimmunity", *Arch. Dermatol.* **119**, 636–640.

Lidor, Y., Topilsky, M., Spitzer, S.A. and Yehoshua, H. (1980) "Autoimmune antibodies in intrinsic (non-atopic) asthma", *Ann. Allergy* **44**, 296–298.

Mackelprang, R., Carlson, C.S., Subrahmanyan, L., Livingston, R.J., Eberle, M.A. and Nickerson, D.A. (2002) "Sequence variation in the human T-cell receptor loci", *Immunol. Rev.* **190**, 26–39.

Marsh, D.G. (1997) "Approaches toward the genetic analysis of complex traits: asthma and atopy", *Am. J. Respir. Crit. Care Med.* **156**, S133–S138.

Matsunaga, Y., Kawasaki, H. and Terada, T. (1999) "Stromal mast cells and nerve fibers in various chronic liver diseases: relevance to hepatic fibrosis", *Am. J. Gastroenterol.* **94**, 1923–1932.

McHugh, R.S. and Shevach, E.M. (2002) "The role of suppressor T cells in regulation of immune responses", *J. Allergy Clin. Immunol.* **110**, 693–702.

Mitsuyasu, H., Izuhara, K., Mao, X.Q., Gao, P.S., Arinobu, Y., Enomoto, T., Kawai, M., Sasaki, S., Dake, Y., Hamasaki, N., Shirakawa, T. and Hopkin, J.M. (1998) "Ile50Val variant IL4R up-regulates IgE synthesis and associates with atopic asthma", *Nat. Genet.* **19**, 119–120.

Morton, S., Palmer, B., Muir, K. and Powell, R.J. (1998) "IgE and non-IgE mediated allergic disorders in systemic lupus erythematosus", *Ann. Rheum. Dis.* **57**, 660–663.

Nahm, D.H., Shin, M.J., Yim, H., Kang, Y., Choi, D.C., Kim, J.K., Kim, S.S., Lee, S.K. and Park, H.S. (2001) "Increased levels of circulating autoantibodies to cultured human bronchial epithelial cell in adult patients with nonatopic asthma", *J. Korean Med. Sci.* **16**, 407–410.

Nahm, D.H., Lee, Y.E., Yim, E.J., Park, H.S., Yim, H., Kang, Y. and Kim, J.K. (2002) "Identification of cytokeratin 18 as a bronchial epithelial autoantigen associated with nonallergic asthma", *Am. J. Respir. Crit. Care Med.* **165**, 1536–1539.

Pesci, A., Bertorelli, G., Gabrielli, M. and Olivieri, D. (1993) "Mast cells in fibrotic lung disorders", *Chest* **103**, 989–996.

Robbie-Ryan, M. and Brown, M. (2002) "The role of mast cells in allergy and autoimmunity", *Curr. Opin. Immunol.* **14**, 728–733.

Rudwaleit, M., Andermann, B., Alten, R., Sorensen, H., Listing, J., Zink, A., Sieper, J. and Braun, J. (2002) "Atopic disorders in ankylosing spondylitis and rheumatoid arthritis", *Ann. Rheum. Dis.* **61**, 968–974.

Sato, A., Takemura, Y., Yamada, T., Ohtsuka, H., Sakai, H., Miyahara, Y., Aizawa, T., Terao, A., Onuma, S., Junen, K., Kanamori, A., Nakamura, Y., Tejima, E., Ito, Y. and Kamijo, K. (1999) "A possible role of immunoglobulin E in patients with hyperthyroid Graves' disease", *J. Clin. Endocrinol. Metab.* **84**, 3602–3605.

Seiberler, S., Bugajska-Schretter, A., Hufnagl, P., Binder, B.R., Stockl, J., Spitzauer, S., Valent, P. and Valenta, R. (1999) "Characterization of IgE-reactive autoantigens in atopic dermatitis. Subcellular distribution and tissue-specific expression", *Int. Arch. Allergy Immunol.* **120**, 108–116.

Sekigawa, I., Yoshiike, T., Iida, N., Hashimoto, H. and Ogawa, H. (2002) "Allergic disorders in systemic lupus erythematosus: prevalence and family history", *Lupus* **11**, 426–429.

Sperr, W.R., Natter, S., Baghestanian, M., Smolen, J., Wolff, K., Binder, B.R., Muller, M.R., Lechner, K., Valenta, R. and Valent, P. (2000) "Autoantibody reactivity in a case of Schnitzler's syndrome: evidence for a Th1-like response and detection of IgG2 anti-Fc$\epsilon$RI$\alpha$ antibodies", *Int. Arch. Allergy Immunol.* **122**, 279–286.

Stevens, R.L. and Austen, K.F. (1989) "Recent advances in the cellular and molecular biology of mast cells", *Immunol. Today* **10**, 381–386.

Szczeklik, A., Nizankowska, E., Serafin, A., Dyczek, A., Duplaga, M. and Musial, J. (1995) "Autoimmune phenomena in bronchial asthma with special reference to aspirin intolerance", *Am. J. Respir. Crit. Care Med.* **152**, 1753–1756.

Tan, E.M., Feltkamp, T.E., Smolen, J.S., Butcher, B., Dawkins, R., Fritzler, M.J., Gordon, T., Hardin, J.A., Kalden, J.R., Lahita, R.G., Maini, R.N., McDougal, J.S., Rothfield, N.F., Smeenk, R.J., Takasaki, Y., Wiik, A., Wilson, M.R. and Koziol, J.A. (1997) "Range of antinuclear antibodies in healthy individuals", *Arthritis Rheum.* **40**, 1601–1611.

de Vries, J.E. (1998) "The role of IL-13 and its receptor in allergy and inflammatory responses", *J. Allergy Clin. Immunol.* **102**, 165–169.

Wallukat, G. and Wollenberger, A. (1991) "Autoantibodies to beta 2-adrenergic receptors with antiadrenergic activity from patients with allergic asthma", *J. Allergy Clin. Immunol.* **88**, 581–587.

Woolley, D.E. and Tetlow, L.C. (2000) "Mast cell activation and its relation to proinflammatory cytokine production in the rheumatoid lesion", *Arthritis Res.* **2**, 65–74.

Zurawski, G. and de Vries, J.E. (1994) "Interleukin 13, an interleukin 4-like cytokine that acts on monocytes and B cells, but not on T cells", *Immunol. Today* **15**, 19–26.

